# Neuroprotective Effects of Safflower Flavonoid Extract in 6-Hydroxydopamine-Induced Model of Parkinson’s Disease May Be Related to its Anti-Inflammatory Action

**DOI:** 10.3390/molecules25215206

**Published:** 2020-11-09

**Authors:** Hui Lei, Rutong Ren, Yi Sun, Ke Zhang, Xin Zhao, Nuramatjan Ablat, Xiaoping Pu

**Affiliations:** 1 Department of Molecular and Cellular Pharmacology, School of Pharmaceutical Sciences, Peking University, Beijing 100191, China; leihui2006sdu@sina.cn (H.L.); sunyi@bjmu.edu.cn (Y.S.); zhangke6880@163.com (K.Z.); zhaoxin2010@bjmu.edu.cn (X.Z.); nuramatjan817@bjmu.edu.cn (N.A.); 2 Department of Pharmaceutical Chemistry, Shandong Qidu Pharmaceutical Co., Ltd., Zibo 255400, China; rrt874609@163.com

**Keywords:** safflower flavonoid extract, Parkinson’s disease, 6-OHDA, mouse model, anti-inflammatory activities

## Abstract

Safflower (*Carthamus tinctorius. L.*), a Chinese materia medica, is widely used for the treatment of cardiovascular and cerebrovascular diseases, with flavonoids being the major active components. Multiple flavonoids in safflower bind to Parkinson’s disease (PD)-related protein DJ-1. Safflower flavonoid extract (SAFE) improved behavioral indicators in a 6-hydroxydopamine (6-OHDA)-induced rat model of PD; however, the underlying mechanisms remain unclear. We used a 6-OHDA-induced mouse model of PD and a primary neuron-astrocyte coculture system to determine the neuroprotective effects and mechanisms of SAFE. After three weeks of SAFE administration, behavioral indicators of PD mice were improved. SAFE regulated the levels of tyrosine hydroxylase (TH) and dopamine metabolism. It significantly inhibited the activation of astrocytes surrounding the substantia nigra and reduced Iba-1 protein level in the striatum of PD mice. SAFE reduced the plasma content of inflammatory factors and suppressed the activation of nod-like receptor protein 3 (NLRP3) inflammasome. In the coculture system, kaempferol 3-O-rutinoside and anhydrosafflor yellow B significantly improved neuronal survival, suppressed neuronal apoptosis, and reduced IL-1β and IL-10 levels in the medium. Thus, SAFE showed a significant anti-PD effect, which is mainly associated with flavonoid anti-inflammatory activities.

## 1. Introduction

Parkinson’s disease (PD), also known as tremor paralysis, is one of the most common neurodegenerative diseases [1]. The main pathological features of PD are gradual loss of nigrostriatal dopaminergic neurons, appearance of characteristic Lewy bodies in the dense part of the substantia nigra (SN), decrease in dopamine (DA) release in the nigrostriatal striatum pathway, and significant reduction in striatum DA [2].

Neuroinflammation has been reported to play a crucial role in the pathogenesis of PD and other neurodegenerative diseases [3]. Knott et al. found that astrocytes and microglia were activated in the brain of patients with PD [4]. In-depth study of the mechanism of interaction between astrocytes, microglia, and neurons is of great significance for the treatment of PD [5]. Nucleotide-binding domain leucine-rich repeat-containing receptor family (or Nod-like receptor protein family), pyrin domain-containing 3 (NLRP3) inflammasome participates in the cascade of downstream inflammatory factors by activating cysteine-containing aspartate proteolytic enzyme 1 (caspase 1) and regulating the differentiation and maturation of upstream proinflammatory factors, such as interleukin (IL)-1β [6]. The inflammatory cascade induced after the activation of NLRP3-caspase-1 inflammatory body is related to the progression of PD [7].

Current treatment methods for PD include surgery and, mainly, drug treatment [8]. Early PD treatment strategies focused on improving DA deficiency, which caused abnormal movement in patients. Levodopa and DA agonists can ameliorate motor function at all stages of PD but are most effective in the early stages and often lead to severe non-motor side effects, including psychiatric symptoms [9]. The monoamine oxidase-B inhibitors selegiline and rasagiline are administered to delay and reduce the need for future use of levodopa or combined with levodopa to lessen motor fluctuating symptoms and “off” seizures in patients with advanced PD [10]. Deep brain stimulation as a surgical therapy is effective to treat motor complications that cannot be satisfactorily controlled with medical therapy [11]. Although existing methods can alleviate PD symptoms, they are limited in fundamentally preventing or reversing the course of the disease [12]. Therefore, PD pathogenesis and effective treatment methods to prevent and delay the pathological process of PD are major medical research topics.

Flavonoids are the main components of safflower that exert pharmacological effects, including antioxidative, anticoagulation, anti-inflammatory, and neuroprotective effects [13]. In previous studies, we found that the main components of safflower flavonoid extract (SAFE), kaempferol 3-O-rutinoside (K3R) and anhydrosafflor yellow B (AYB), could bind with the PD-related protein DJ-1 [14]. Additionally, we isolated a standardized SAFE and prepared drop pills [15,16]. SAFE can improve behavioral and biochemical and pathological changes in 1-methyl-4-phenyl-1,2,3,6-tetrahydropyridine-induced PD mice and rotenone- or 6-hydroxydopamine (OHDA)-induced PD rats [15,17,18]. The number of glial fibrillary acidic protein (GFAP)-positive astrocytes in the SN of 6-OHDA-induced PD rats was increased, suggesting that 6-OHDA may have caused inflammation in rat brains. In this study, we aimed to further investigate whether SAFE treatment regulates the PD inflammatory process and elucidate the possible underlying mechanisms.

## 2. Results

### 2.1. Safflower Flavonoid Extract (SAFE) Improves Behavioral Dysfunction in Parkinson’s Disease (PD) Mice

**Figure 1:**
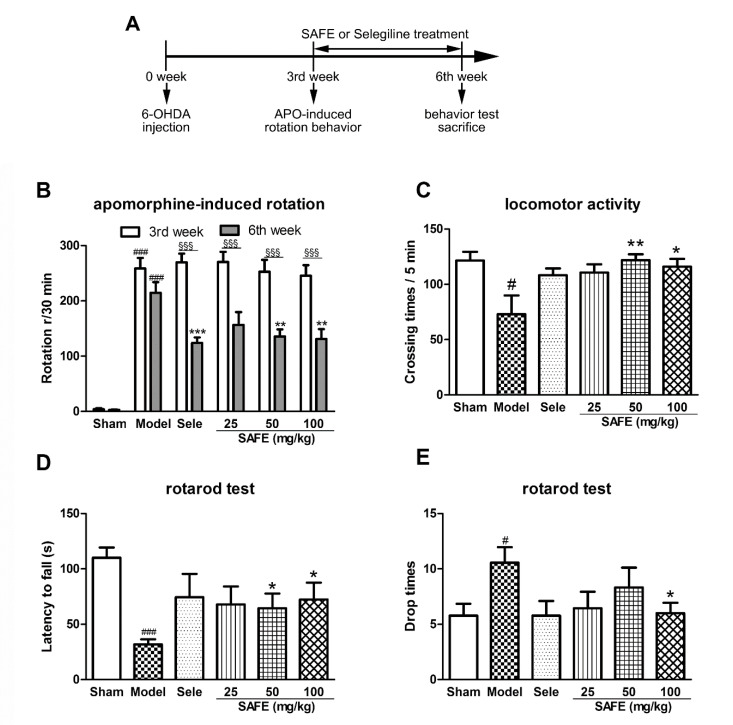
(**A**) Flow chart of the experimental setup. Effect of safflower flavonoid extract (SAFE) on motor function of 6-OHDA-induced Parkinson’s disease (PD) mice. (**B**) Apomorphine-induced rotational behavior of mice at the 3rd week and 6th week; (**C**) Locomotor activity was assessed using a mouse autonomous activity instrument at the 6th week; (**D**) Latency to fall represents time from start to first fall from rod on rotarod test at the 6th week; (**E**) Drop times of mice represent the number falls from the rod within 180 s on the rotarod test at the 6th week. *n* = 9. Sele, selegiline 15 mg/kg control group. ^#^ *p* < 0.05, ^###^ *p* < 0.001 vs. sham group. * *p* < 0.05, ** *p* < 0.01, *** *p* < 0.001 vs. model group and ^§§§^ *p* < 0.001 each group at 6th week vs. that at 3rd week.

The effect of SAFE on the motor function of PD mice was evaluated using apomorphine-induced rotation, autonomous activity, and rotarod tests. At the sixth week, the rotations after apomorphine induction markedly increased (Figure 1B, *p* < 0.001), crossing times in the autonomous activity test significantly reduced (Figure 1C, *p* < 0.05), latent period to fall in the rotarod test significantly shortened (Figure 1D, *p* < 0.001), and the time of rod-dropping significantly increased (Figure 1E, *p* < 0.05) in the 6-OHDA model group as compared with the sham group.

After three weeks of administration (at the sixth week), animal apomorphine-induced rotations of each group were significantly reduced as compared with the third week (Figure 1B, *p* < 0.001). The rotations of SAFE groups significantly decreased as compared with those of the model group (50 mg/kg, 100 mg/kg, *p* < 0.01, Figure 1B). Compared with the model group, the crossing number of autonomous activities of SAFE groups was significantly increased (50 mg/kg, *p* < 0.01 and 100 mg/kg, *p* < 0.05, Figure 1C), the latent period to fall in the rotarod test was prolonged (50 mg/kg and 100 mg/kg, *p* < 0.05, Figure 1D), and the time of dropping was significantly reduced (100 mg/kg, *p* < 0.05, Figure 1E).

### 2.2. SAFE Reduces Tyrosine Hydroxylase (TH) Level in the Substantia Nigra (SN) of Mice

**Figure 2:**
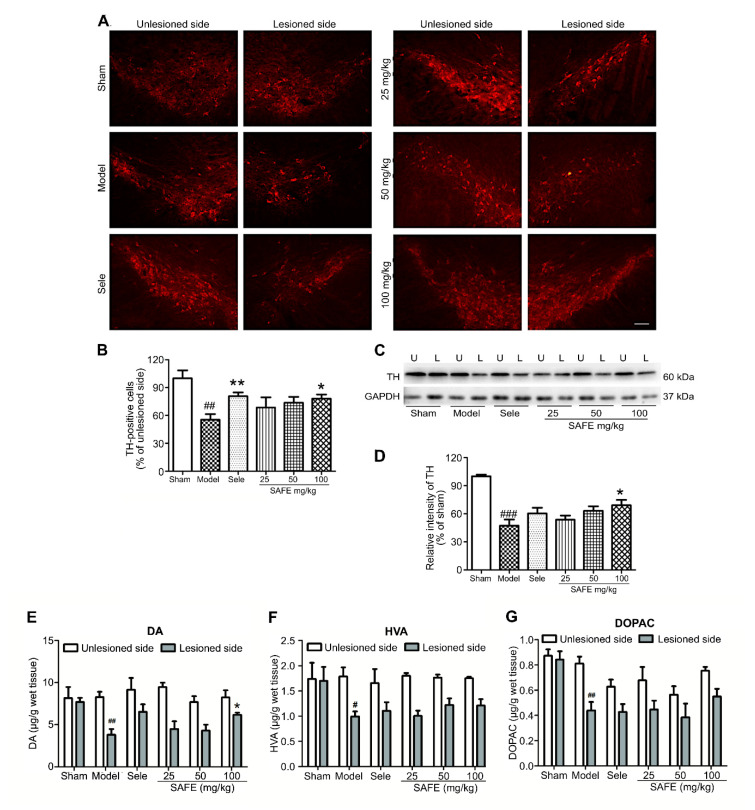
Effect of SAFE on dopamine metabolism. (**A**) Representative photographs of tyrosine hydroxylase (TH)-stained sections (bar = 100 μm) in the substantia nigra (SN); (**B**) Ratios of TH-positive cells numbers in the lesioned SN to those in the unlesioned SN of each group, *n* = 5; (**C**) Representative Western blot for TH protein in the SN. U, unlesioned side and L, lesioned side; (**D**) Quantitative analysis of the relative intensity of TH/GAPDH in lesioned side of each group as compared with that of the sham group, *n* = 3; Contents of dopamine (DA) (**E**), homovanillic acid (HVA) (**F**), and dihydroxyphenyl acetic acid (DOPAC) (**G**), respectively, in the striatum measured by high-performance liquid chromatography coupled with electrochemical detection (HPLC-ECD), *n* = 4. ^#^ *p* < 0.05, ^##^ *p* < 0.01, ^###^ *p* < 0.001 vs. sham group and * *p* < 0.05, ** *p* < 0.01 vs. model group.

The number of tyrosine hydroxylase (TH)-positive cells in the lesioned side of the SN was significantly reduced in the model group as compared with the sham group (Figure 2B, *p* < 0.01). SAFE at 100 mg/kg induced a significant increase in TH-positive cells in the SN as compared with the model group (*p* < 0.05, Figure 2B). TH protein expression in the SN was significantly reduced in the model group as compared with that in the sham group (Figure 2D, *p* < 0.001) and that of the 100 mg/kg SAFE group was significantly increased as compared with the expression in the model group (Figure 2D, *p* < 0.05).

### 2.3. SAFE Regulates Dopamine (DA) Metabolism

Changes in DA, homovanillic acid (HVA), and dihydroxyphenyl acetic acid (DOPAC) contents in the striatum of PD mice were detected by high-performance liquid chromatography coupled with electrochemical detection (HPLC-ECD). Compared with the sham group, the model group showed significantly reduced DA (Figure 2E, *p* < 0.01), HVA (Figure 2F, *p* < 0.05), and DOPAC (Figure 2G, *p* < 0.01). Compared with the model group, the 100 mg/kg SAFE group showed a significant increase in DA content in the striatum (Figure 2E, *p* < 0.05).

### 2.4. SAFE Suppresses the Activation of Astrocytes and Microglia

**Figure 3:**
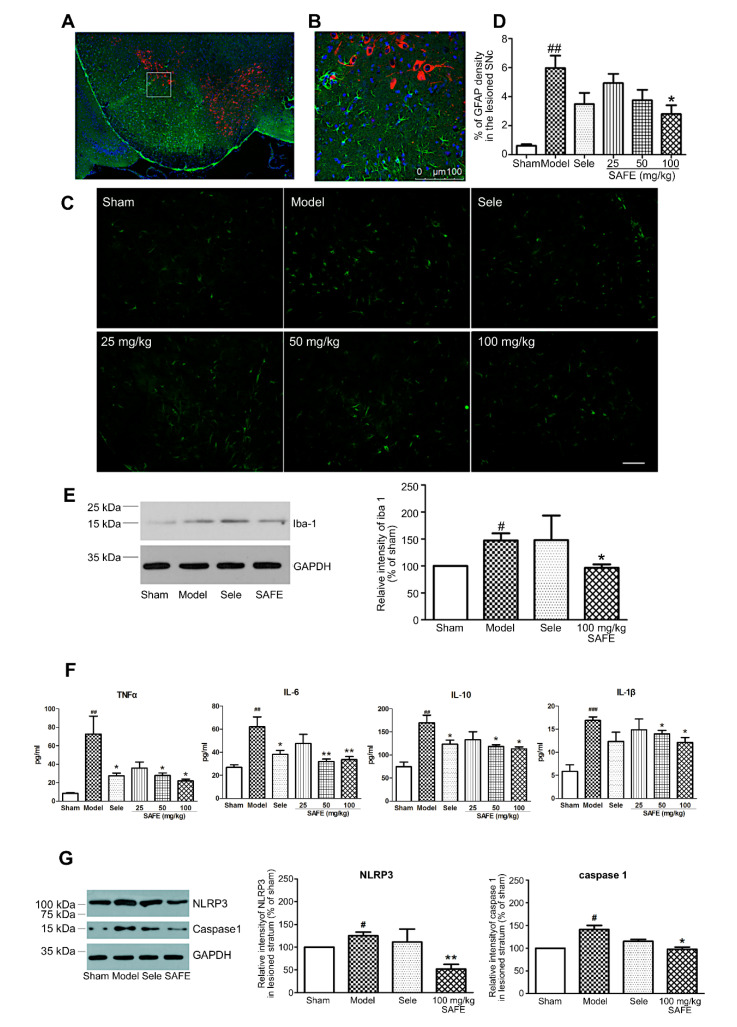
Effect of SAFE on inflammation. (**A**) Representative photographs of co-staining (model group, coronal section) of TH (red), glial fibrillary acidic protein (GFAP, green), and DAPI (blue) in the SN; (**B**) Enlarged views of the indicated regions in (A); (**C**) Representative images of GFAP-stained cells in the SN of mice in each group of animals (bar = 50 μm); (**D**) Fluorescence intensity levels of GFAP-stained cells in the lesioned side of the SN in each group, *n* = 4; (**E**) Representative Western blot and quantitative analysis of the relative intensity of Iba-1/GAPDH in the striatum, *n* = 3; (**F**) Plasma contents of TNF-α (*n* = 7), IL-6 (*n* = 8), IL-10 (*n* = 5), and IL-1β (*n* = 4). Cytokine concentrations were determined using ELISA kits; (**G**) Representative Western blot images and quantitative analysis of the relative intensity of NLRP3/GAPDH and caspase 1/GAPDH, *n* = 3. ^#^ *p* < 0.05, ^##^ *p* < 0.01, ^###^ *p* < 0.001 vs. sham group and * *p* < 0.05, ** *p* < 0.01 vs. model group.

Immunofluorescence staining results showed that astrocytes clustered around TH-positive cells with large cell bodies and thick protrusions, indicating that they were in an activated state (Figure 3A–C). On the basis of the area of astrocyte clusters/total area, we found that 100 mg/kg SAFE significantly suppressed astrocyte activation (Figure 3D, *p* < 0.05).

Western blotting results revealed that Iba-1 protein level in the striatum of PD mice was increased (Figure 3E, *p* < 0.05), and this increase was significantly suppressed in the 100 mg/kg SAFE group (Figure 3E, *p* < 0.05).

### 2.5. SAFE Reduces the Levels of Inflammatory Factors

The plasma contents of inflammatory factors TNF-α (*p* < 0.01), IL-6 (*p* < 0.01), IL-10 (*p* < 0.01), and IL-1β (*p* < 0.001) in the model group were significantly increased. The SAFE treatment (50 and 100 mg/kg) suppressed the increase in TNF-α (*p* < 0.05), IL-6 (*p* < 0.01), IL-10 (*p* < 0.05), and IL-1β (*p* < 0.05) (Figure 3F).

### 2.6. SAFE Inhibits NLRP3 Inflammasome Activation

Western blotting results showed that the expression levels of NLRP3 and caspase 1 in the striatum were significantly increased (Figure 3G, *p* < 0.05). The SAFE (100 mg/kg) treatment suppressed the increase in NLRP3 (*p* < 0.01, Figure 3G) and caspase 1 (*p* < 0.05, Figure 3G).

### 2.7. K3R and AYB Play a Protective Role in a Coculture System

**Figure 4:**
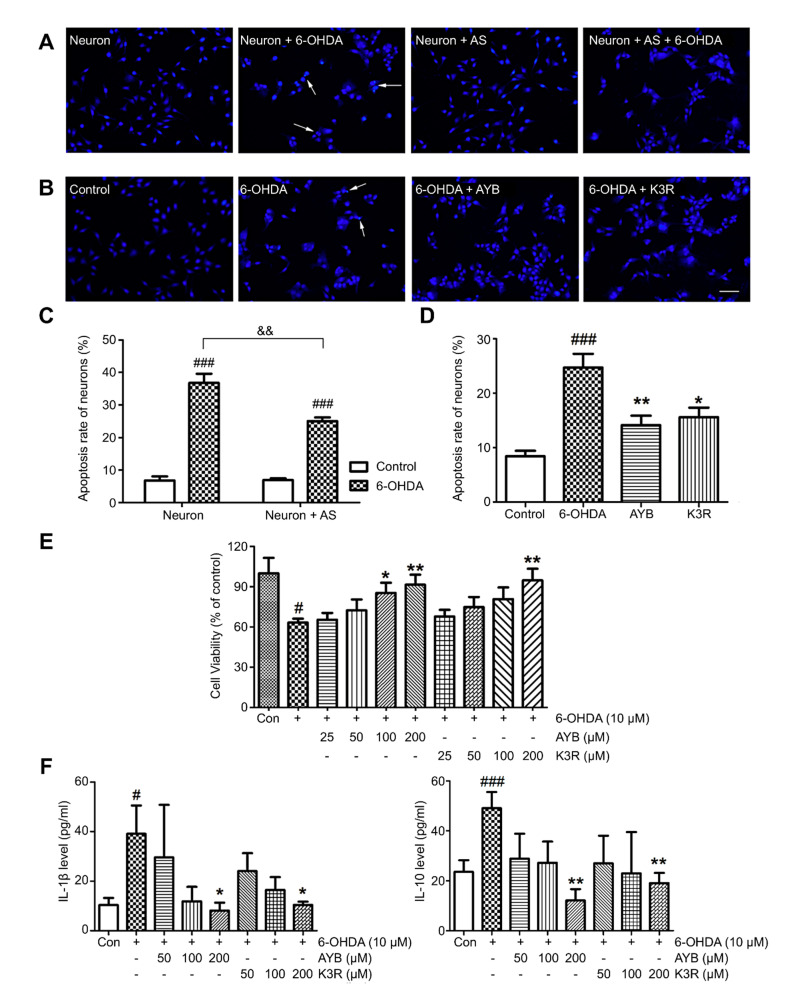
Effect of K3R and AYB in a neuron-astrocyte coculture system. (**A**–**C**) The coculture (neuron + astrocyte (AS)) system reduced apoptosis rate of neurons as compared with single-culture (neuron). Representative photographs of nuclear bodies stained with Hoechst 33342, apoptotic cell nuclei were indicated by the arrows; (**B**–**D**), Effect of K3R and SYB on the neuronal apoptosis rate in the coculture system (bar = 50 μm); (**E**) Effects of K3R and AYB on the neuronal viability in the coculture system damaged by 6-OHDA; (**F**) Contents of IL-1β and IL-10 in the co-culture medium. *n* = 6. ^#^ *p* < 0.05, ^###^ *p* < 0.001 vs. control group; * *p* < 0.05, ** *p* < 0.01 vs. 6-OHDA group; and ^&&^ *p* < 0.01 6-OHDA group of co-culture vs. 6-OHDA group of single culture.

A neuron-astrocyte coculture system was established, and Hoechst 33342 fluorescence staining was performed to investigate neuronal apoptosis after 6-OHDA injury (the final concentration, 10 μM and the incubation time, 24 h). Normal cell nucleus showed uniform elliptical or circular blue fluorescence, whereas apoptotic cell nucleus showed concentrated half-moon, lobulated, or fragmented fluorescence (as indicated by the arrow in Figure 4A). Single-cultured and cocultured neurons showed significant apoptosis after 6-OHDA injury. The apoptosis rate of neurons in single culture with 6-OHDA injury increased from 6.8% to 36.8% (*p* < 0.001), whereas that of neurons in the coculture system increased from 6.9% to 25.0% (*p* < 0.001). Compared with neurons cultured alone, neurons in the coculture system showed a significant decrease in apoptosis rate (Figure 4A–C, *p* < 0.01), suggesting that astrocytes play a protective role in neuronal apoptosis after 6-OHDA injury.

Next, 100 μM AYB or K3R was added to the coculture system for 2 h; then, 10 μM 6-OHDA was added to induce injury for 24 h, and Hoechst 33342 fluorescence staining was performed to detect apoptosis. Apoptosis rate in the normal control group in the coculture system was 8.4 ± 1.0% and that of the 6-OHDA model group was 24.7 ± 2.5%, which was significantly higher (*p* < 0.001) (Figure 4B, D). AYB or K3R (both 100 μM) significantly reduced neuronal apoptosis rate to 14.1 ± 1.7% (*p* < 0.01) and 15.6 ± 1.7% (*p* < 0.05), respectively.

Neuronal survival rate in the 6-OHDA model group (63.2 ± 2.9%) was lower than that in the control group (*p* < 0.05) (Figure 4E). AYB at 100 or 200 μM significantly increased cell survival rate. K3R administration at 200 μM significantly increased the cell survival rate to 94.8 ± 8.5% (*p* < 0.01).

IL-1β and IL-10 contents in the coculture medium were detected by ELISA (Figure 4F). IL-1β and IL-10 contents in the control group were 10.4 ± 2.9 pg/mL and 23.6 ± 4.7 pg/mL, respectively. After 6-OHDA induction, they significantly increased to 39.1 ± 11.4 pg/mL (*p* < 0.05) and 49.1 ± 6.5 pg/mL (*p* < 0.001), respectively. AYB and K3R (both 200 μM) significantly reduced the contents of IL-1β (AYB, 8.1 ± 3.2 pg/mL, *p* < 0.05 and K3R, 10.4 ± 1.3 pg/mL, *p* < 0.05) and IL-10 (AYB, 12.1 ± 4.5 pg/mL, *p* < 0.01 and K3R, 19.1 ± 4.0 pg/mL, *p* < 0.01).

## 3. Discussion

After three weeks of administration of different doses of SAFE, the number of apomorphine-induced rotations of the mice was significantly reduced, the crossing times of autonomous activities were greatly increased, the latency to fall in the rod test was extended, and the number of drops was significantly reduced as compared with that in the model group. These are consistent with our previous results on 1-methyl-4-phenyl-1,2,3,6-tetrahydropyridine-induced PD mice and rotenone- or 6-OHDA-induced PD rats [15,17,18]. These results indicated that SAFE can improve 6-OHDA-induced motor dysfunction in PD mice.

TH is an enzyme that limits DA synthesis in the brain, and its content directly affects DA biosynthesis [19]. TH immunofluorescence staining can directly reflect the severity of 6-OHDA-injured dopaminergic neurons and effects of drug intervention. In the TH immunofluorescence staining experiment, the ratio of TH-positive cells on the lesioned side to that on the unlesioned side of the SN in the high-dose SAFE group was significantly increased. Western blotting results of TH protein level in the lesioned side are in agreement with this finding. Reduction in DA and its metabolites is the main neurobiochemical change in PD [20]. Therefore, DA and its metabolites in the striatum are used as one of the main indicators for the clinical diagnosis of PD and are the most objective indicators to evaluate drug effectiveness. The HPLC-ECD results showed that the contents of DA, DOPAC, and HVA in the striatum were significantly regulated by SAFE treatment. In a rat PD model induced by rotenone, SAFE showed similar effects [17]. These effects of SAFE on TH and DA indicate that it can potentially be used in PD therapy. Additionally, in the primary neuron-astrocyte coculture system, both AYB and K3R significantly increased the viability and decreased the apoptosis rate of neurons. Similarly, AYB and K3R had significant effects on cell viability and microtubule stability in rotenone-induced PC12 cells [15]. These results suggested that SAFE was beneficial to the survival of dopaminergic neurons.

Although the etiology and pathogenesis of PD are complex and have not been fully elucidated, neuroinflammation plays a vital role during the course of PD [21]. Under stimulation (e.g., by cerebral injury, neuroinflammation, and causative agents), microglia are activated and subsequently deliver substantial neurotoxic substances, such as TNF-α, IL-1β, and IL-18, which then work together to trigger neurodegeneration [22]. In addition, astrocytes are involved in the pathogenesis of PD and have important physiological and pathological significance. Post-mortem autopsy of patients have revealed an increase in the number of astrocytes and pathological changes in their specific distribution patterns [23]. In this study, we found that astrocytes in the periphery of the SN and microglia in the striatum exhibited different levels of activation after 6-OHDA unilateral striatum injection, and the plasma contents of inflammatory cytokines were significantly increased. After SAFE administration, glial activation and inflammatory factor levels were restrained. K3R and AYB significantly suppressed 6-OHDA-induced increases in the contents of IL-1β and IL-10 in the medium of coculture system. Together, these results showed that SAFE lowers inflammatory activity.

The NLRP3 inflammasome is important in the inflammation process of PD [6]. A study by Mao et al. showed that NLRP3 inflammasome was involved in PD pathogenesis and inhibition of NLRP3/caspase-1/IL-1β pathways relieved symptoms, providing an approach to PD prevention and therapy [24]. Chen et al. demonstrated that naringenin, a flavonoid extracted from citrus fruits and grapefruits, produced neuroprotective effects to resist LPS-induced neurological toxicity by inhibiting microglial NLRP3 inflammasome activation [25]. Our studies have come up with similar results, i.e., SAFE was shown to reduce the expression of NLRP3 and caspase 1 proteins.

The pathogenesis and clinical treatment outcomes of PD suggest that it is difficult to slow disease progression using a single drug with a single effect. This natural flavonoid compound with multiple biological functions provides us with new theoretical and experimental bases for the prevention and cure of neurodegenerative disorders. Many similar compounds in traditional Chinese medicine, such as flavonoids, often act on the same or similar targets. The occurrence of disease implies changes in multiple metabolic processes in the body, which leads to the destruction of homeostasis. Traditional Chinese medicine can produce better therapeutic effects than a single target drug by acting on related multiple targets. According to the Drug Administration Law of the People’s Republic of China, the state encourages the use of modern science and technology to research and develop traditional Chinese patent medicines and strengthen the quality control of traditional Chinese medicine. In the announcement on classification of the Chinese medicine registration and filing requirements, effective parts and preparations extracted from plants, animals, minerals, and other substances that have not been put on the market in China can be applied for as new drugs of Chinese medicine and natural products. This is the major advantage of traditional Chinese medicine. More in-depth studies are needed to evaluate the mechanism of SAFE, including studies of the upstream and downstream NLRP3 inflammasome factors and gene expression. In addition, our findings were observed in a single experimental animal model and need to be verified in more in vivo and in vitro models and in clinical trials.

## 4. Materials and Methods

### 4.1. Reagents and Animals

SAFE with standardized quality used in this study was a laboratory-made drip pill prepared by Ren [15,16]. K3R was purchased from Shanghai Nature Standard Corporation (Shanghai, China). AYB was purchased from Chengdu Must Biotechnology (Chengdu, China).

An apomorphine hydrochloride standard (100839-200601) was purchased from the National Institutes for Food and Drug Control (Beijing, China). 6-OHDA, polylysine, and 1-β-d-arabinofuranosylcytosine (Ara-C) were purchased from Sigma-Aldrich (St Louis, MO, USA). Dulbecco’s modified Eagle’s medium (DMEM), DMEM/F12, neurobasal-A medium, B27, and GlutaMAX-I were purchased from Invitrogen (Carlsbad, CA, USA). Mouse TNFα, IL-6, IL-1β, and IL-10 ELISA kits were purchased from Wuhan Boster Biological Technology (Wuhan, China). TH mouse monoclonal antibody (sc-25269) was purchased from Santa Cruz Biotechnology (Delaware Ave Santa Cruz, CA, USA); GFAP mouse mAb (#3670) and GAPDH rabbit mAb (#2118) were purchased from Cell Signaling Technology (Danvers, MA, USA); anti-Iba1 antibody (ab178847) and anti-NLRP3 antibody (ab214185) were purchased from Abcam (Cambridge, UK); anti-caspase1 antibody (YT0652) was purchased from Immunoway Biotechnology Company (Plano, TX, USA). Can Get Signal Immunoreaction Enhancer Solution 1 (NKB-201) was obtained from TOYOBO Biotech support Department (Osaka, Japan).

Eight-week-old C57BL/6 mice (SPF grade) were obtained from Beijing Vital River Laboratory Animal Technology (SCXK (Beijing) 2011-0011, Beijing, China). Neonatal (less than 24 h old) C57BL/6 mice (SPF grade) were obtained from the Department of Laboratory Animal Science of Peking University Health Science Center (SCXK (Beijing) 2016-0010, Beijing, China). All experiments and operating procedures were carried out under the guidance of the Animal Protection Committee of Peking University Health Science Center and approved by Peking University Medical Ethics Committee (no. LA2016245). The experimental setup is shown in Figure 1A.

### 4.2. 6-OHDA-Induced PD Mouse Model Establishment and Verification

To produce more selective destruction of the nigrostriatal dopaminergic pathway, toxin has been injected in subregions of the caudate-putamen complex (CPu) in many recent studies [26]. The mice were allocated to the following six randomized groups: a sham operation control group (sham group, 20 mice); a model control group (model group, 20 mice); a selegiline 15 mg/kg control group (Sele, 20 mice); a low-dose SAFE group (25 mg/kg, 20 mice); a medium-dose SAFE group (50 mg/kg, 20 mice); and a high-dose SAFE group (100 mg/kg, 20 mice). After they were anesthetized, the mice were bound to a stereotaxic instrument (RWD Life Science, Shenzhen, China).

The bregma was the “0” point coordinate, and the mouse brain stereotactic striatum coordinate was set to AP = +0.5 mm, ML = –1.8 mm, and DV = –3.5 mm. The mice in the sham group were administered 2 μL of saline with 0.2% vitamin C, whereas mice in other groups were administered 6-OHDA solution (2 μL, 4 μg/μL, dissolved in saline with 0.2% vitamin C). The injection speed was set to 0.5 μL/min. After the needle was sustained for 3 min, it was slowly redrawn for 2 min.

An apomorphine-induced rotation assessment was used to test the model. A mouse with stable rotation and a speed greater than 5 laps/min was considered to be a successful PD model. The safflower pills were dissolved in physiological saline and administered by gavage every day. After 3 weeks of continuous administration, behavioral experiments, including apomorphine-induced rotation behavior, autonomous activity, and a rotarod test, were conducted.

### 4.3. Apomorphine-Induced Rotation Assessment

Three weeks after injection, the model mice were tested using an apomorphine-induced rotation assessment. Each mouse to be tested was placed in a standard laboratory beaker (height 14 cm and diameter 11.5 cm) and allowed to acclimate for 20 min. Then, the mice were administered subcutaneous injection of apomorphine (0.5 mg/kg) and placed back in the beaker. Immediately thereafter, the turns that the mice made to the healthy side were counted for 30 min, and the data were analyzed to determine the number of net rotations during this period.

### 4.4. Mouse Autonomous Activity

Using a mouse autonomous activity instrument, each mouse was placed in the center of an autonomous activity chamber (height 13 cm and diameter 25 cm) connected to an infrared tracking analyzer that transmitted the activity data of the mouse to a computer. After the mice were acclimated for 2 min, the total number of horizontal and vertical movements within 5 min was recorded.

### 4.5. Rotarod Test

The roller rotation speed was set to 35 r/min, and each mouse was placed on the rotarod after the rotation speed was reached. The test lasted for 180 s. The time from the moment the mouse was placed on the wheel surface to the first slip and the number of slips within 180 s were recorded. The time from start to first fall from the rod was recorded as the latency to fall, and the number of times the mouse fell within 180 s was recorded as the falling number. If the mouse still did not fall at 180 s, it was recorded as 180 s. Each mouse was tested three times, and each measurement was separated by 30 min; the average was taken for statistical analysis.

### 4.6. Brain Sampling and Sectioning

After the behavioral experiment, the mice were anesthetized, and blood was collected by cardiac puncture. Then, the brains were perfused with 20–30 mL Tris-buffered saline (TBS), soaked in 4% paraformaldehyde for 2.5 h, and incubated in graded sucrose (10%, 20%, and 30%, prepared with TBS) at 4 °C. Coronal slices were prepared using a cryostat (CM 1950, IL 60089 United States Leica biosystems, Buffalo Grove, IL USA) and used for immunofluorescence staining.

### 4.7. Immunofluorescence

The sections were blocked in 10% sheep serum (diluted in phosphate-buffered saline (PBS) containing 0.5% Triton X-100) for 60 min to reduce non-specific adsorption. Then, the sections were incubated overnight with appropriately diluted primary antibody (prepared with antibody diluent, TH 1:100, GFAP 1:200) at 4 °C. After rinsing with PBS, the sections were incubated with fluorescent-labeled secondary antibodies (Alexa Fluor 555 goat anti-rabbit IgG and Alexa Fluor 488 goat anti-mouse IgG, Invitrogen, 1:200 ratio dilution) at 37 °C for 60 min. Then, the sections were rinsed, mounted on glass slides, and imaged under a confocal fluorescence microscope (Leica TCS SP8, Leica Microsystems, Buffalo Grove, IL, USA).

### 4.8. Detection of Dopamine and Its Metabolites in the Striatum

The contents of DA, HVA, and DOPAC in striatum tissue were detected using an electrochemical detector (LC-4B, BASi, West Lafayette, IN, USA). Next, the tissue sample were weighed, mixed with 0.6 M HClO_4_ in a ratio of 1:10, to prepare a tissue homogenate by ultrasonic disruption (200 w, 8 s, 10-s interval, 3 times), and centrifuged at 10,000× *g* at 4 °C for 20 min. Then, the supernatant was mixed with Solution B (1:0.5, containing 20 mM potassium citrate, 300 mM potassium dihydrogen phosphate, EDTA·Na_2_·2H_2_O 2 mM) well and sat quietly, and then centrifuged for 20 min. The supernatant was aspirated and centrifuged again for 20 min. The final supernatant was store at 4 °C for test. Sodium citrate buffer (pH 3.68) containing 85 mM citric acid, 0.1 M anhydrous sodium acetate, 15% methanol, and 0.2 mM Na_2_ EDTA was used as mobile phase. The Ag/AgCl reference electrode was used, and the detection potential was set at 0.76 V. The flow rate was 1.2 mL/min at 25 °C.

### 4.9. Primary Cell Culture and Coculture System

Cell culture was conducted, as described previously [27]. For primary culture of mouse astrocytes, striatum, and SN tissues were isolated from newborn mice within 24 h, minced, and digested with 0.25% trypsin for 20 min in a water bath at 37 °C. After centrifugation (120× *g*, 5 min, 37 °C), the pelleted cells were suspended in DMEM/F12 containing 10% fetal bovine serum in polylysine-coated flasks and incubated at 37 °C. The entire medium was changed after 24 h; then, every 3 days, half of the medium was renewed. After approximately 12 days, when astrocytes covered the bottom of the culture flask, they were digested with 0.25% trypsin for 3–5 min, collected, and transferred (4 × 10^5^ cells/mL) into the upper chamber of a Transwell system (#3460, 0.4 μm, Corning Inc., Corning, NY, USA).

For primary culture of mouse neurons, striatum and SN tissues of neonatal mice were collected, minced, and digested with 0.125% pancreatin at 37 °C for 10 min to obtain neurons. The pelleted cells were resuspended in DMEM with 10% horse serum and 10% fetal bovine serum, and then transferred (4 × 10^5^ cells/mL) into the lower chamber of a Transwell plate coated with polylysine. Four hours later, the medium was changed to neurobasal-A medium containing 2% B27. After 48 h, 10 μM Ara-C was added, and the entire solution was changed at 72 h to discard Ara-C. The cells were cultured for 7 days for use in experiments.

When neurons were cultured until the fifth day as described above, the upper Transwell chamber containing the astrocyte layer was inserted above the lower Transwell chamber containing neurons, and the medium was changed to neurobasal-A with 2% B27 and 0.5 mM GlutaMAX to form a neuron-astroglial coculture system. Experiments were conducted after 3 days of cocultivation. Morphological changes of apoptosis in neurons were studied by Hoechst 33342 staining. Cells were injured by 6-OHDA with the final concentration 10 μM for 24 h. Then Hoechst 33342 (10 mg/l) was added to the cells and incubated for 10 min at 37 °C in the dark. The neurons were washed twice by the serum-free medium, and then were examined using a fluorescence microscopy. The percentage of the apoptotic neurons was calculated by the ratio of apoptotic neurons to the total neurons counted.

### 4.10. Western Blotting

Mouse SN tissues were disrupted by ultrasonic disruption (200 w, 8 s, 10 s interval, 3 times). Proteins were quantified by the bicinchoninic acid method, separated by sodium dodecyl sulfate-polyacrylamide gel electrophoresis, and transferred to a polyvinylidene difluoride membrane. The membrane was blocked with TBS containing 5% skimmed milk powder for 2 h, hybridized with primary antibody (TH 1:500, Iba-1 1:500, NLRP3 1:1000, caspase 1, 1:1000, GAPDH, 1:500) at 4 °C overnight, and incubated with the secondary antibody at 37 °C for 2 h. Then, Immobilon Western Chemilum HRP Substrate was added, and the gel was imaged (ChemiDoc XRS System, Bio-Rad Laboratories, Hercules, CA, USA) for immunocomplex detection. Quantity One software (Bio-Rad Laboratories, Hercules, CA, USA) was used to quantify the gray level of protein bands in the image.

### 4.11. Statistical Analysis

All data are presented as mean ± standard error of the mean (SEM) and analyzed using SPSS 16.0 software (IBM, Armonk, NY, USA). Differences between groups were evaluated using one-way analysis of variance followed by least significant difference post hoc tests; *p* < 0.05 was deemed significant.

## 5. Conclusions

In conclusion, our results demonstrate that SAFE exerts neuroprotective effects on 6-OHDA-induced dyskinesia and dopaminergic neuron degeneration in PD mice. Notably, SAFE can reduce the secretion of inflammatory factors via the attenuation of microglial NLRP3 inflammasome activation. This study suggests that SAFE is a potential drug for PD treatment.
